# Cycle training modulates satellite cell and transcriptional responses to a bout of resistance exercise

**DOI:** 10.14814/phy2.12973

**Published:** 2016-09-20

**Authors:** Kevin A. Murach, R. Grace Walton, Christopher S. Fry, Sami L. Michaelis, Jason S. Groshong, Brian S. Finlin, Philip A. Kern, Charlotte A. Peterson

**Affiliations:** ^1^ Department of Rehabilitation SciencesCenter for Muscle BiologyCollege of Health SciencesUniversity of KentuckyLexingtonKentucky; ^2^ Department of Nutrition and MetabolismUniversity of Texas Medical BranchGalvestonTexas; ^3^ Division of Endocrinology, and Barnstable Brown Diabetes and Obesity CenterDepartment of MedicineUniversity of KentuckyLexingtonKentucky

**Keywords:** Acute exercise, aerobic training, cytokines, fiber type‐specific

## Abstract

This investigation evaluated whether moderate‐intensity cycle ergometer training affects satellite cell and molecular responses to acute maximal concentric/eccentric resistance exercise in middle‐aged women. Baseline and 72 h postresistance exercise *vastus lateralis* biopsies were obtained from seven healthy middle‐aged women (56 ± 5 years, BMI 26 ± 1, VO_2max_ 27 ± 4) before and after 12 weeks of cycle training. Myosin heavy chain (MyHC) I‐ and II‐associated satellite cell density and cross‐sectional area was determined via immunohistochemistry. Expression of 93 genes representative of the muscle‐remodeling environment was also measured via NanoString. Overall fiber size increased ~20% with cycle training (*P* = 0.052). MyHC I satellite cell density increased 29% in response to acute resistance exercise before endurance training and 50% with endurance training (*P* < 0.05). Following endurance training, MyHC I satellite cell density decreased by 13% in response to acute resistance exercise (acute resistance × training interaction, *P* < 0.05). Genes with an interaction effect tracked with satellite cell behavior, increasing in the untrained state and decreasing in the endurance trained state in response to resistance exercise. Similar satellite cell and gene expression response patterns indicate coordinated regulation of the muscle environment to promote adaptation. Moderate‐intensity endurance cycle training modulates the response to acute resistance exercise, potentially conditioning the muscle for more intense concentric/eccentric activity. These results suggest that cycle training is an effective endurance exercise modality for promoting growth in middle‐aged women, who are susceptible to muscle mass loss with progressing age.

## Introduction

Endurance cycle training appears to result in some of the same responses as resistance training in untrained muscle. Our laboratory (Fry et al. 2014) and others (McCarthy et al. 2002; McPhee et al. 2010; Harber et al. 2012; Konopka and Harber 2014) have shown that moderate‐intensity cycle ergometer training has anabolic potential and can promote skeletal muscle hypertrophy. Moreover, the anabolic qualities of cycle ergometer training are pronounced in older women (Sillanpaa et al. 2009; Hudelmaier et al. 2010; Konopka et al. 2010; Harber et al. 2012). Hypertrophy from cycle training is unique because endurance adaptations occur simultaneously with resistance‐type adaptations within the same mode. Moreover, endurance cycle training is primarily concentric, submaximal, and noninjurious whereas resistance training is usually concentric/eccentric, maximal, and initially damaging. While adaptive responsiveness to a bout of familiar exercise is documented in well‐trained skeletal muscle (Coffey et al. 2006a; Murach et al. 2014), the global cellular responses to a given exercise type are generally less pronounced and more targeted as conditioning within that mode improves (Schmutz et al. 2006; Perry et al. 2010; Raue et al. 2012; Egan et al. 2013; Nader et al. 2014; Damas et al. 2016). A recent explanation for this phenomenon is that the initial responses to exercise (within 1 week) globally reflect muscle damage and tissue repair while later responses (3 weeks and beyond) are more nuanced and “refined” for promoting specific training outcomes (e.g., hypertrophy) (Damas et al. 2016). However, it is unclear how adaptations (hypertrophic or otherwise) induced by primarily concentric, moderate‐intensity cycle training affect responses to an unfamiliar acute maximal concentric/eccentric resistance exercise bout.

It is well‐established that satellite cell pool expansion is characteristic of the resistance exercise‐mediated hypertrophic process in humans (Crameri et al. 2004, 2007; Dreyer et al. 2006; Petrella et al. 2006; O'Reilly et al. 2008; McKay et al. 2009; Mikkelsen et al. 2009; Babcock et al. 2012; Bellamy et al. 2014; Farup et al. 2014; Hyldahl et al. 2014; Snijders et al. 2014). In this investigation, we evaluated fiber type‐specific satellite cell density following unaccustomed maximal concentric/eccentric exercise before and after 12 weeks of moderate‐intensity concentric cycle training in middle‐aged women. The women in this investigation overlap with those from a previous investigation, most of which demonstrated increased MyHC I and overall satellite cell density as well as global muscle fiber hypertrophy in response to cycle training (Fry et al. 2014). We hypothesized that concentric endurance training would condition the muscle for a maximal concentric/eccentric exercise bout by modulating the satellite cell response. Furthermore, we evaluated the expression of genes that are indicative of the global muscle‐remodeling environment that may also influence satellite cell behavior (i.e., angiogenesis, cytokine/trophic factor, extracellular matrix, growth/remodeling, immunity/inflammation, and metabolism‐related genes) (Walton et al. 2015). We hypothesized that molecular responses to acute resistance exercise would also be attenuated after cycle training and would track with satellite cell behavior, signaling improved conditioning in an unaccustomed exercise mode among these middle‐aged women.

## Methods

### Subjects

Seven inactive nonobese women classified as nondiabetic from an oral glucose tolerance test were included in this analysis (Table 1). Subjects generally did not have a history of any type of exercise training, although one subject did engage in recreational endurance activity at the time of study admission. All women were peri‐ or postmenopausal, and the subjects presented here overlap with a larger cohort evaluated previously for angiogenic and satellite cell responses to endurance training (Fry et al. 2014; Walton et al. 2015). However, the effects of endurance training on acute resistance exercise responses in the subjects presented here have not previously been reported. Exclusion criteria included: history of smoking, coronary disease, congestive heart failure, chronic inflammatory diseases, trigylcerides >700 mg/dL, or orthopedic problems that could limit participation in the exercise protocols. Subjects also were not taking any medications known to affect skeletal muscle biology, such as angiotensin‐converting enzyme inhibitors, angiotensin II receptor blockers, statins, steroids, or anti‐inflammatory drugs. Subjects were instructed to maintain consistent dietary and lifestyle habits throughout the investigation. All subjects were informed of the design and purpose of the study prior to signing consent forms. All procedures were approved by the Institutional Review Board of the University of Kentucky and performed in accordance with the standards set forth by the Declaration of Helsinki.

**Table 1:** Subject characteristics

Characteristic	Mean ± SD	Range
Age (years)	56 ± 5	51–64
Body mass index (kg/m^2^)	26.2 ± 1.0	24.3–27.5
Body mass (kg) untrained	70.8 ± 5.3	62.1–79.4
Body mass (kg) endurance trained	69.6 ± 5.4^a^	60.8–79.4
VO_2max_ (mL/kg/min) untrained	26.5 ± 4.0	19.0–31.7
VO_2max_ (mL/kg/min) endurance trained	30.6 ± 6.1^a^	21.6–42.6
VO_2max_ (L/min) untrained	1.8 ± 0.2	1.5–2.2
VO_2max_ (L/min) endurance trained	2.1 ± 0.3^a^	1.7–2.6

a *P* < 0.05 untrained versus endurance trained.

### Study design

**Figure 1:**
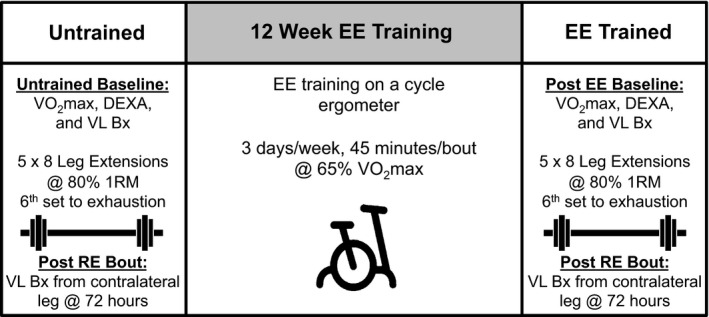
Overview of study design. 1RM, one repetition maximum; Bx, muscle biopsy; DEXA, dual energy X‐ray absorptiometry; EE, endurance exercise; RE, resistance exercise; VO_2max_, maximal aerobic capacity; VL,* vastus lateralis*.

An overview of the study design is shown in Figure 1. After screening, subjects underwent a basal dual energy X‐ray absorptiometry (DEXA, Lunar Prodigy; GE Lunar Inc., Little Chalfont, U.K.) scan as well as a percutaneous skeletal muscle biopsy of the *vastus lateralis*, using a Begström 5 mm needle with suction (Bergström 1962). All subjects arrived at the clinic in the fasted state and completed a maximal oxygen consumption (VO_2max_) graded exercise test on a bicycle ergometer (Monark Sports and Medical, Stockholm, Sweden) to evaluate pretraining aerobic capacity. Heart rate, blood pressure, and rating of perceived exertion were monitored. Ventilation and expired air samples were measured by a metabolic cart (Vmax Encore Metabolic Cart; CareFusion, San Diego, CA) for the determination of O_2_ uptake. Following baseline testing, subjects completed an acute bout of resistance exercise followed by a 72 h postresistance exercise biopsy from the opposite leg of the basal biopsy. Subjects then endurance trained on a bicycle ergometer for 12 weeks. After 12 weeks of training, subjects repeated the DEXA and VO_2max_ testing. A basal biopsy was taken 72 h following the last endurance training bout in order to capture the state of chronically trained muscle and avoid any effects of detraining. Acute resistance exercise was repeated in the trained state, followed by another 72 h postresistance exercise biopsy. Gene expression measures 72 h after resistance exercise temporally match the satellite cell measures, are consistent with previous investigations from our laboratory utilizing the same postexercise time point (Dennis et al. 2008, 2009), and capture the gene responses associated with muscle inflammation, cytokines, and adaptive remodeling after unaccustomed exercise that appear to have a protracted time course (Louis et al. 2007; Neubauer et al. 2014).

### Acute resistance exercise and chronic endurance training

For the acute resistance exercise bout, subjects performed 10 min of light cycling as a warm‐up. Each subject's one repetition maximum (1 RM) was determined for the leg extension (Keiser Pneumatic Strength Training Equipment; Keiser, Fresno, CA) on the leg contralateral to the baseline biopsy during a familiarization trial performed at screening and again after endurance training. Subjects performed five sets of eight repetitions and a sixth set to volitional fatigue on the leg extension, all at 80% 1 RM. Resistance exercise was performed on the leg contralateral to the baseline biopsy. Two minutes of rest was allotted between each set, and verbal encouragement was provided during each RE bout. This protocol was chosen because it represents a lower extremity exercise paradigm aimed at increasing muscle mass and strength. After baseline testing, 12 weeks of aerobic exercise training was carried out 3 days/week for 45 min per session at an intensity corresponding to 65% VO_2max_. Training intensity was monitored throughout via heart rate monitor (Polar Electro Inc., Woodbury, NY) and adjusted as fitness improved. Subjects were allowed to take brief breaks at the beginning of training but were able to complete the entire 45‐min session continuously at a heart rate corresponding to 65% VO_2max_ by the eighth week and through the conclusion of training.

### Immunohistochemistry

Five of the seven subjects presented here were part of a larger investigation in which myosin heavy chain (MyHC) fiber type distribution (MyHC I, I/IIa, IIa, and IIa/IIx) and cross‐sectional area were presented (Fry et al. 2014). Of the seven, satellite cell data for one subject was not included as tissue was no longer available for analysis at one time point. For the six remaining subjects, fiber type‐specific satellite cell density was analyzed as previously described (Fry et al. 2014). Muscle tissue samples (~50 mg) were mounted in tragacanth gum on cork immediately after the biopsy and frozen in liquid‐nitrogen cooled isopentane. Seven micrometer sections were cut, dried for 1 h, and fixed in ice‐cold acetone for 3 min. Following a phosphate buffered solution (PBS) wash, primary antibodies for MyHC I (1:75, BA.D5; DSHB, Iowa City, IA) and laminin (1:100, L9393; Sigma‐Aldrich, St. Louis, MO) diluted in PBS were applied overnight at 4°C. Endogenous peroxidases were blocked with 3% H_2_O_2_, sections were washed in PBS, and then incubated for 1 h at room temperature with secondary antibodies (1:250, goat anti‐mouse IgG2b alexa fluor 647, #A21242, and 1:250, goat anti‐rabbit IgG alexa fluor 488, #A11034; Invitrogen, Carlsbad, CA) in PBS. Following another PBS wash, sections were blocked for 1 h in 2.5% normal horse serum (S‐2012; Vector, Burlingame, CA) and incubated overnight in primary antibody for Pax7 (1:100, PAX7; DSHB) diluted in 2.5% normal horse serum while rocking at 4°C. The next day, sections were washed in PBS and TSA signal amplification was performed with AlexaFluor 594 (T20935; Invitrogen) according to the manufacturer protocol. After a final wash in PBS, sections were postfixed for 5 min in methanol and mounted, using Vectashield with DAPI (H1200; Vector).

On separate sections, fiber type and fiber type‐specific cross‐sectional area in the untrained and endurance trained state were determined as previously described (Fry et al. 2014). Briefly, 7 *μ*m sections were incubated for 90 min at room temperature in antibodies against MyHC I, MyHC IIa (neat, IgG1, SC.71), and MyHC IIx (neat, IgM, 6H1) from DSHB and rabbit anti‐laminin (1:100) from Sigma‐Aldrich. After PBS wash, sections were incubated in isotype specific anti‐mouse secondary antibodies for MyHC I, MyHC IIa (1:500, IgG1 alexa fluor 488, #A21121), and MyHC IIx (1:250, IgM alexa fluor 555, #A21426) from Invitrogen, all diluted in PBS, along with the secondary antibody for laminin (1:150, IgG AMCA, CI‐1000; Vector). Sections were postfixed and mounted, using Vectashield (H1000; Vector).

### Image acquisition and analysis

Images for analysis were captured at 20× magnification, using the Zeiss AxioImager M1 microcope (Zeiss, Oberkochen, Germany). Analysis was performed, using Zen digital imaging software (Zeiss). Fiber type‐specific satellite cell density was assessed using a Pax7 antibody in conjunction with antibodies against MyHC I (fiber type), laminin (fiber borders), and DAPI (nuclei). Cells within the laminin border and positive for both Pax7 and DAPI were counted as satellite cells within each given fiber type. A minimum of 150 fibers per subject was used for fiber type and fiber type‐specific size analysis. For satellite cell analysis, an average of 333 (range 183–631) muscle fibers and 12 satellite cells (range 8–45) per biopsy at each time point were identified. A total of 8002 muscle fibers and 567 satellite cells were included in the analysis.

### NanoString gene expression analysis

As previously described by our laboratory (Walton et al. 2015), RNA was extracted from muscle biopsy samples from all four time points for all seven subjects by homogenizing pulverized frozen samples in QIAzol Lysis Reagent (79306; QIAGEN, Hilden, Germany). RNA was precipitated and washed using the RNeasy kit (74104; QIAGEN). RNA quality and integrity was assessed using the Agilent 2100 Bioanalyzer (Agilent Technologies, Santa Clara, CA). Gene expression was measured using the highly‐sensitive nCounter analysis system (NanoString Technologies, Seattle, WA) (Kulkarni 2011). As previously described, we designed a hypothesis‐driven custom probe set that spanned 109 genes in angiogenesis, cytokine/trophic factor, extracellular matrix, growth/remodeling, immunity/inflammation, and metabolism‐related processes (Walton et al. 2015). The nCounter code set was hybridized with 100 ng of RNA from each biopsy. Data were normalized according to NanoString's instructions. Briefly, a positive control normalization was conducted to account for platform‐associated sources of variation, the background of the average of negative controls was subtracted, and the geometric mean of six reference genes (b‐actin, Cyclophilin A, Cyclophilin B, TATA‐binding protein, Tubulin‐b, and Ubiquitin C) was used to calculate a normalization factor that was applied to all genes in a given sample. Raw housekeeping gene geometric means were not significantly different across time points. All data are presented as normalized counts.

### Statistics

Data were checked for normality and log transformations were applied prior to analysis of non‐normal data. Normal data were otherwise used for analysis. In order to evaluate how training affected satellite cells per fiber, muscle fiber size, fiber type distribution, and subject characteristics, unidirectional paired *t*‐tests were employed using SPSS (IBM, Armonk, NY). Directional *t*‐tests were employed based on the previous findings of Fry et al. (2014). In order to evaluate changes in gene expression and satellite cells per area following endurance training, bidirectional paired *t*‐tests were performed using SPSS. To evaluate whether endurance training influenced the satellite cell and gene response to resistance exercise, two‐way repeated measures ANOVAs were employed using SPSS. Significance was set at *P* < 0.05. Data are presented as mean ± standard deviation unless otherwise denoted.

## Results

### Subject characteristics, fiber size, and fiber type distribution

Table 1 shows changes in aerobic capacity and body mass with endurance training. The reduction in body fat percentage with cycle training (−2.3%) approached significance (*P* = 0.10, data not shown) and the reduction in body mass (−1.7%) achieved significance (*P* < 0.05). Relative (mL/kg/min) and absolute (L/min) VO_2max_ was increased by 16% and 17%, respectively, after the 12 week training period (*P* < 0.05). These changes were greater in magnitude compared to those reported in the larger overlapping cohort that included men and women encompassing a broad age range and more diverse demographics (Bagley 2014; Fry et al. 2014). As a result of endurance cycle training, fiber cross sectional area of all fibers increased (3571 *μ*m^2^ to 4313 *μ*m^2^, *P* = 0.052), while MyHC I (3756 *μ*m^2^ to 4430 *μ*m^2^, *P* = 0.058) and MyHC IIa (3472 *μ*m^2^ to 4183 *μ*m^2^, *P* = 0.073) fiber cross‐sectional area tended to increase. MyHC IIa percentage tended to increase with cycle training (31% to 38%, *P* = 0.056), while MyHC I (53% to 49%, *P* = 0.07) and MyHC IIa/IIx (14% to 11%, *P* = 0.11) tended to decrease.

### Fiber type‐specific and overall satellite cell responses to acute resistance exercise and endurance exercise training

**Figure 2:**
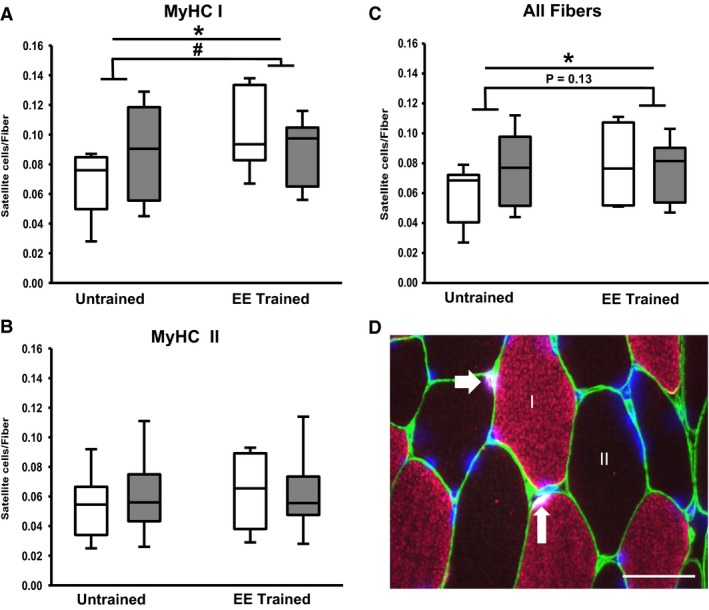
Changes in satellite cell density following endurance exercise training and in response to acute resistance exercise. Quantification of individual satellite cell density changes in myosin heavy chain (MyHC) I (A), II (B), and all fibers (C). White boxes are resting condition and gray boxes are after acute resistance exercise. Panel D is a representative image of fiber type‐specific satellite cell analysis. White arrows point to satellite cells (white) within the laminin border (green) in an MyHC I (pink) and II (unstained) fiber. DAPI (blue) stains all nuclei. Scale bar represents 50 *μ*m. EE, endurance exercise training. *P < 0.05 (endurance training), ^#^ *P* < 0.05 (acute resistance × endurance training interaction).

Figure 2 shows satellite cell changes in response to acute resistance exercise, and the effects of endurance training on that response. Following a bout of resistance exercise, MyHC I satellite cell density increased by 29% (0.068 ± 0.021 to 0.088 ± 0.031 satellite cells per fiber) in the untrained state but declined by 13% in the endurance trained state (0.102 ± 0.025 to 0.089 ± 0.020 satellite cells per fiber, acute resistance × training interaction, *P* < 0.05). Satellite cell density in all fibers showed a trend for a similar pattern of satellite cell response as MyHC I fibers, but the interaction effect for satellite cell density in all fibers did not reach significance (*P* = 0.13). Similar to what was reported in an overlapping cohort (Fry et al. 2014), MyHC I satellite cell density increased by 50% after endurance training (0.068 ± 0.021 to 0.102 ± 0.025 satellite cells per fiber, *P* < 0.05) and satellite cell density in all fibers increased by 32% (0.060 ± 0.018 to 0.079 ± 0.023 satellite cells per fiber, *P* < 0.05). MyHC II satellite cell density did not change with acute resistance exercise or endurance training. After endurance training, the number of satellite cells per muscle fiber cross‐sectional area tended to increase in MyHC I fibers (19.6 ± 6.5 to 26.5 ± 7.7 satellite cells per mm^2^, *P* = 0.097), and remained stable in MyHC II fibers (17.2 ± 6.9 vs. 17.6 ± 7.1 satellite cells per mm^2^, *P* > 0.05) as well as all fibers (17.9 ± 5.9 vs. 21.1 ± 7.1 satellite cells per mm^2^, *P* > 0.05).

### Gene expression responses to acute resistance exercise and endurance exercise training

**Figure 3:**
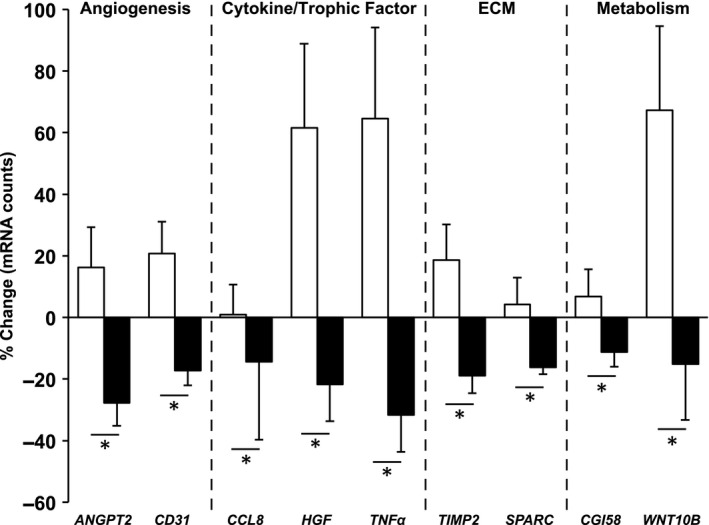
Genes that showed opposite responses to resistance exercise in the untrained and endurance trained states. White bars represent the untrained response and black bars represent the endurance trained response to a bout of resistance exercise, presented as mean ± SE. ECM, extracellular matrix. *P < 0.05 (acute resistance × endurance training interaction).

Ten genes in the probe set demonstrated differential expression 3 days following the resistance exercise bout in the untrained state versus endurance trained state (Fig. 3, Table S1). There was some overlap (i.e., *ANGPT2*,* CD31*,* TNFα*, and *GPIHPB1*) between genes that showed an interaction effect and genes that were modulated by endurance training alone (*P* < 0.05, Table S1). The list of all 93 genes expressed above the detection limit and their responses to resistance exercise and endurance training is found in Table S1. In response to the resistance exercise bout, angiogenesis (*ANGPT2*,* CD31*), cytokine/trophic factor (*CCL8*,* HGF*,* TNFα*), extracellular matrix (*SPARC*,* TIMP2*), and metabolism (*CGI58*,* WNT10B*)‐related genes increased in the untrained state but decreased in the endurance trained state (acute resistance × endurance training interaction, *P* < 0.05 Fig. 3). Other cytokine/trophic factor (*IL12β*), metabolism (*ATGL*,* PPARγ2*,* UCP2*), and immunity/inflammation (*CD163*)‐related genes had the same pattern of expression but did not achieve significance (*P* < 0.10). Only one gene that had an interaction effect (*GPIHPB1*) did not demonstrate this pattern, decreasing expression in both the untrained and endurance trained state (Table S1).

## Discussion

In line with other reports in humans (Babcock et al. 2012; Bellamy et al. 2014; Farup et al. 2014), satellite cell density increased in MyHC I fibers (29%) following a bout of unaccustomed maximal resistance exercise in the untrained state. However, the 50% increase in MyHC I satellite cell density resulting from 12 weeks of endurance training countered the satellite cell proliferative response to acute resistance exercise that was observed before cycle training. Similar patterns of satellite cell and molecular activity with acute resistance exercise signal a less stressful muscle milieu after training and provide further evidence that the cellular microenvironment influences satellite cell behavior (Fuchs et al. 2004; Christov et al. 2007; Yin et al. 2013). Habitual submaximal concentric endurance activity appears to condition satellite cell and molecular responses for a maximal concentric/eccentric exercise stimulus. These data endorse the prescription of cycle training for middle‐aged women since it confers robust cardiovascular benefits while simultaneously serving as a sarcopenia countermeasure.

### Endurance training modulates satellite cell and molecular responses to a bout of resistance exercise

The lack of satellite cell increase following a bout of resistance exercise in the endurance trained state was likely accounted for by the significant 50% increase in resting satellite cell density caused by endurance training. It is conceivable that resistance exercise in the untrained state is more challenging to MyHC I fibers and necessitates satellite cell proliferation for adaptation. Once MyHC I fibers have adjusted to the demands of concentric cycle training and experience a marked increase in resting satellite cell density, acute satellite cell proliferation after maximal concentric/eccentric exercise may become unnecessary or redundant. The lack of satellite cell response in MyHC II fibers with acute resistance exercise or endurance training is contrary to some other findings (Verney et al. 2008; Verdijk et al. 2009; Babcock et al. 2012; Joanisse et al. 2013; Bellamy et al. 2014; Farup et al. 2014), but could be attributable to the unique subject population (middle‐aged women) and/or the nature of the exercise stimuli in this investigation.

Some angiogenesis (*ANGPT2* and *CD31*) and metabolism (*CGI58* and *WNT10B*) genes increased 72 h after resistance exercise when untrained but decreased when endurance trained. *ATGL* also demonstrated this response pattern (*P* < 0.10) and *GPIHPB1* was down‐regulated with resistance exercise after endurance training, collectively suggesting altered lipid metabolism. Thus, adaptations to endurance exercise likely ameliorated the need for resistance‐exercise mediated angiogenic and metabolic responses. Interestingly, endothelial cells (CD31^+^) can regulate satellite cell behavior (Christov et al. 2007), and *CD31* gene expression after resistance exercise tracks with MyHC I satellite cell density. The transcriptional pattern of other genes encoding various cytokines that could affect satellite cell behavior similarly reflected MyHC I satellite cell responses to resistance exercise. TNF*α* and HGF strongly stimulate satellite cell proliferation (Allen et al. 1995; Tatsumi et al. 1998), and the ~60% increase (untrained) and ~20% decrease (endurance trained) after resistance exercise closely follows MyHC I satellite cell responses. Attenuated expression of the chemokine CCL8 (Henningsen et al. 2011) and the secreted regenerative marker SPARC (Jorgensen et al. 2009; Petersson et al. 2013) after endurance training suggests mitigated muscle stress with acute resistance exercise. Likewise, expression of TIMP2, which increases in a damage‐dependent fashion at the gene and protein level after unaccustomed exercise (Koskinen et al. 2001), was elevated with resistance exercise when untrained but decreased after endurance training. Collectively, these data provide further evidence of a complementary relationship between the global cellular environment and satellite cell behavior (Fuchs et al. 2004; Christov et al. 2007; Yin et al. 2013).

### Overlap of genes affected by resistance exercise and endurance training in middle‐aged women

Only three genes that demonstrated opposite responses after resistance exercise in the untrained and endurance trained state had higher resting expression after endurance training (*ANGPT2*,* CD31*,* TNFα*). It is possible that increased baseline levels of these three genes contributed to reduced expression 72 h after resistance exercise when endurance trained. We previously reported robust skeletal muscle and vascular remodeling at the phenotypic and molecular level, including increased *ANGPT2* and *CD31* expression, in an overlapping cohort (Walton et al. 2015). Elevated *TNFα* after endurance training could signal an inflammatory state (Lang et al. 2003; Tidball 2005). However, exercise training typically results in an anti‐inflammatory skeletal muscle milieu (Petersen and Pedersen 2005). A significant baseline increase in anti‐inflammatory cytokine IL‐4 mRNA, as well as no change in classic inflammatory cytokines such as IL‐1*β*, IL‐6, IL‐8, IL‐15, and IL‐18 strongly suggests resting inflammatory processes are not negatively impacted by endurance training. All other genes that responded divergently 72 h after resistance exercise in the untrained and endurance trained state were not affected by endurance training alone. Thus, training‐induced baseline differences do not solely account for differential responses to a bout of resistance exercise.

### Perspectives and summary

In humans, satellite cell pool size and function reportedly predicts hypertrophic potential with resistance training (Petrella et al. 2006, 2008). Thus, reduced satellite proliferation with resistance exercise after cycle training could mean that hypertrophy may be blunted with continued resistance training. However, this conclusion would be debatable since: (1) endurance training itself increased satellite cell density and elicited hypertrophy at the muscle fiber level in these middle‐aged women, (2) combined endurance and resistance (concurrent) training can produce greater hypertrophy versus resistance training alone (Lundberg et al. 2013, 2014; Kazior et al. 2016; Murach and Bagley 2016), (3) robust and unconstrained muscle fiber hypertrophy can proceed in the presence of markedly increased muscle oxidative potential (Scheffler et al. 2014; Omairi et al. 2016), and (4) highly cycle‐trained muscle mounts an early anabolic signaling response after unfamiliar acute resistance exercise (Coffey et al. 2006b). While speculative, it is conceivable that satellite cell adaptations resulting from endurance training could in some way facilitate the exaggerated hypertrophic response sometimes observed with concurrent training (Murach and Bagley 2016), and may have a positive effect on adaptation if resistance or concurrent training was undertaken after cycle training. In general, cycle training may be the preferred mode of endurance training to curtail progressive muscle loss with age. This is especially true for middle‐aged women that do not elect to engage in resistance training. Collectively, the satellite cell and gene expression data presented here indicate that moderate‐intensity endurance cycle training modulates the response to acute resistance exercise, potentially conditioning the muscle for more intense concentric/eccentric activity. These data also provide further evidence that the cellular microenvironment influences satellite cell behavior in humans.

## Conflict of Interest

The authors have no conflicts to declare.

## Supporting information




**Table S1.** NanoString data: targeted gene expression changes in response to endurance cycle training and acute resistance exercise.
